# The Efficiency of FLAIR Images for Hemodynamic Change After STA-MCA Bypass with Moyamoya Disease and Symptomatic Steno-Occlusive Disorder

**DOI:** 10.3390/jcm14103292

**Published:** 2025-05-08

**Authors:** Hyun Dong Yoo, Seung Young Chung, Seong Min Kim, Ki Seok Park, Seung Jun Ryu, Jae Guk Kim

**Affiliations:** Department of Neurosurgery and Neurology, Eulji University Hospital, College of Medicine, Eulji University, Daejeon 35233, Republic of Korea; 20190107@eulji.ac.kr (H.D.Y.); nsksm@eulji.ac.kr (S.M.K.); ks3432@eulji.ac.kr (K.S.P.); spinesj@eulji.ac.kr (S.J.R.); jgkim@eulji.ac.kr (J.G.K.)

**Keywords:** hyperintense vessels, FLAIR MRI, moyamoya disease, STA-MCA bypass, cerebrovascular reserve, SPECT, ICA/MCA steno-occlusion, hemodynamic improvement

## Abstract

**Background**: Hyperintense vessels (HVs) visualized on FLAIR MRI are believed to reflect sluggish antegrade or retrograde flow in leptomeningeal collaterals that develop in response to major intracranial artery stenosis or occlusion. HV is frequently observed in conditions such as Moyamoya disease and symptomatic ICA/MCA steno-occlusion. However, the relationship between HV and cerebral hemodynamics—and the effect of STA-MCA bypass on HV—remains inadequately characterized. This study aimed to investigate the relationship between HV on FLAIR and cerebral vascular hemodynamic status, as measured by SPECT, in patients with Moyamoya disease and symptomatic ICA/MCA occlusion. The secondary goal was to assess the impact of recanalization through STA-MCA bypass surgery on the presence of HV. **Methods**: We retrospectively analyzed 49 patients with symptomatic ICA or MCA steno-occlusion who underwent STA-MCA bypass between 2015 and 2020. Pre- and postoperative FLAIR MRIs were evaluated, and HV presence was graded as negative (0), minimal (1), or positive (2). SPECT was utilized to assess cerebrovascular reserve (CVR) in regions exhibiting various HV intensities. Follow-up FLAIR imaging was performed 3–14 months postoperatively to correlate HV changes with hemodynamic improvements observed via SPECT. **Result**: HV was present in 74% (36/49) of affected hemispheres. Regions exhibiting minimal or positive HV demonstrated a significantly lower CVR compared to HV-negative areas, indicating compromised perfusion. Following bypass surgery, HV was reduced or resolved in 65% (32/49) of patients, and this regression corresponded with improved CVR as confirmed by both SPECT and perfusion MRI. **Conclusions**: HV presence on FLAIR imaging is associated with impaired cerebrovascular hemodynamics in patients with Moyamoya disease or symptomatic large-vessel steno-occlusion. HV-positive territories exhibit reduced CVR, while surgical revascularization via STA-MCA bypass leads to hemodynamic improvement and concurrent HV reduction. These findings support HV as a potential surrogate marker for treatment response.

## 1. Introduction

Hyperintense vessels (HVs) observed on FLAIR MRI resemble diffuse leptomeningeal enhancement patterns—such as the ivy sign and spaghetti sign—typically seen on post-contrast imaging [1]. On MR fluid-attenuated inversion recovery (FLAIR) sequences, HV is described as continuous linear hyperintensities along the leptomeningeal surfaces, particularly following the cortical sulci and within the subarachnoid space [2]. These hyperintense signals are frequently seen on the cortical surface and are strongly correlated with reduced cerebral vascular reserve (CVR) [3,4]. As a result, recent studies have investigated FLAIR imaging as a non-invasive modality to monitor hemodynamic status following superficial temporal artery–middle cerebral artery (STA–MCA) bypass in patients with symptomatic steno-occlusive disease and Moyamoya disease [4]. The ivy sign on unenhanced FLAIR imaging is believed to reflect decreased cerebral perfusion, engorgement of the pial vasculature, and sluggish flow through leptomeningeal collaterals. A robust pial arterial network frequently emerges over the cortical surface after STA–MCA anastomosis, suggesting active neovascularization. These findings support the interpretation that FLAIR hyperintensities represent evolving collateral pathways, especially in Moyamoya disease, where leptomeningeal anastomoses serve a critical compensatory role. Given the retrograde and sluggish nature of such collateral flow, this engorgement is likely to appear hyperintense on contrast-enhanced or FLAIR images. HV appears as focal, tubular, or serpentine high-signal structures within the subarachnoid space, clearly distinguishable from the normally low-signal cerebrospinal fluid (CSF). These features are considered indirect indicators of impaired cerebral hemodynamics. Unlike previous studies that merely described the presence or extent of the ivy sign, our study adopts a semi-quantitative, region-specific HV grading system and correlates it with pre- and postoperative SPECT perfusion data to evaluate hemodynamic changes more precisely.

## 2. Materials and Methods

### 2.1. Patients

Between January 2015 and February 2020, a total of 49 patients (mean age: 51.3 years; range: 23–64 years) underwent STA–MCA bypass surgery at our institution. Of these, 17 were diagnosed with Moyamoya disease and 32 with symptomatic steno-occlusive disease. All patients had preoperative and postoperative MR FLAIR imaging. The decision to perform STA–MCA bypass was based on a combination of clinical symptoms and advanced imaging findings. Surgical candidates were required to have significant intracranial arterial stenosis or occlusion confirmed by angiography or high-resolution MR imaging, along with evidence of impaired cerebral perfusion—typically demonstrated by a diminished cerebrovascular reserve (CVR) on SPECT or perfusion MRI. Recurrent neurological symptoms unresponsive to medical management, including transient ischemic attacks (TIAs) or strokes, further supported surgical indication. A favorable vascular anatomy, including a suitable superficial temporal artery and appropriate recipient vessels within the MCA territory, was also considered essential for surgical eligibility. Contraindications to surgery included severe systemic illness (e.g., advanced cardiac, pulmonary, or renal disease), advanced age with poor physiological reserve, or diffuse vascular pathology involving multiple major cerebral arteries beyond the MCA or ACA territory. Additional exclusions included active infections (particularly in the head or neck), pronounced cerebral atrophy affecting areas of irreversible damage, and a poor likelihood of compliance with postoperative management such as antiplatelet therapy and routine follow-up. Radiation sensitivity and anesthesia-related risks posed by preoperative imaging studies, including SPECT, were also considered relative contraindications. In summary, STA–MCA bypass was performed only in patients who met specific clinical, anatomical, and hemodynamic criteria. Careful candidate selection was prioritized to maximize surgical efficacy while minimizing associated risks.

### 2.2. Imaging Studies

All patients underwent preoperative and postoperative neuroimaging, including MRI, CT angiography, and single-photon emission computed tomography (SPECT). The MRI protocol included both perfusion-weighted imaging and fluid-attenuated inversion recovery (FLAIR) sequences. All images were independently reviewed by a board-certified neuroradiologist, who evaluated and scored the presence of the ivy sign. The cortico-subcortical regions of each cerebral hemisphere were divided into four vascular territories for regional analysis: the anterior cerebral artery (ACA), the anterior portion of the middle cerebral artery (ant-MCA), the posterior portion of the middle cerebral artery (post-MCA), and the posterior cerebral artery (PCA). The central sulcus was used as an anatomical landmark to differentiate the ant-MCA and post-MCA regions. HV on FLAIR imaging was assessed using a semi-quantitative, three-tiered scoring system:

Grade 0 (negative): No visible hyperintense ivy sign;
Grade 1 (minimal): Mild hyperintensity with an unclear or faint ivy sign;
Grade 2 (positive): Clearly visible linear or punctate high signal intensity in the subarachnoid space.

For SPECT, perfusion maps were visualized using a 20-level color scale ranging from black to white, with color intensities graded in 5-point increments (0–100). Red hues corresponding to intensity values of 60–80 represented adequate cerebral perfusion. All SPECT scores were independently verified by the neuroradiologist based on consistent color thresholds. The cerebrovascular reserve (CVR) was evaluated before and after acetazolamide administration. For the purposes of this study, only post-acetazolamide CVR values were used, as they are strongly correlated with the risk of stroke recurrence in patients with symptomatic cerebral ischemia. Pre- and postoperative comparisons of CVR and FLAIR-based HV scores were performed to investigate the correlation between radiological hyperintensity and cerebral hemodynamic status following STA–MCA bypass.

### 2.3. Statistical Analysis

Statistical analyses were performed using Student’s *t*-tests to evaluate the association between HV scores on FLAIR imaging and cerebrovascular reserve (CVR) as measured by SPECT. A *p*-value of less than 0.05 was considered statistically significant. Paired Student’s *t*-tests were used to compare pre- and postoperative HV and CVR values within the same vascular territories of individual patients. To mitigate the intra-subject correlation from multiple regional measurements per hemisphere, regional averages were calculated. Given the limited sample size and retrospective nature of this study, mixed-effects models were not applied.

## 3. Results

A total of 49 patients were diagnosed with Moyamoya disease or symptomatic steno-occlusive disease based on angiographic findings, and all underwent STA–MCA bypass surgery. The sum of cerebrovascular reserve (CVR) values derived from post-contrast SPECT imaging increased from 3035 preoperatively to 3480 postoperatively, demonstrating a statistically significant improvement following surgery (*p* < 0.05). To illustrate the regional hemodynamic variation, Table 1 summarizes the HV scores and estimated CVR values across the major vascular territories—including the ACA, anterior MCA, posterior MCA, and PCA—before and after STA–MCA bypass.

**Figure 1:**
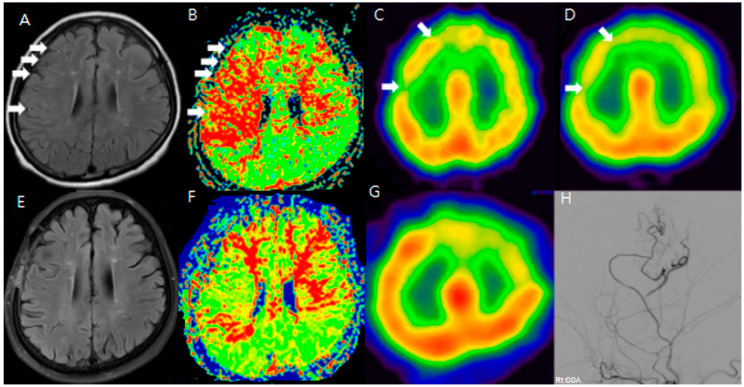
F/64 Moyamoya disease patients. Preoperative (**A**–**D**) and postoperative (**E**–**H**) MR FLAIR, perfusion MR, and SPECT show improved HV and CVR after STA-MCA bypass (white arrow: hyperintense vessels and decreased CVR in the same region). The areas showing hyperintense vessels on FLAIR (white arrows in (**A**)) correspond to zones of diminished CVR on SPECT (**B**–**D**). After STA-MCA bypass, the reduction in HV (**E**) is associated with improved perfusion, suggesting that the ivy sign reflects dynamic cerebrovascular status (**F**–**H**).

Among these patients, MR FLAIR and SPECT images were checked after the anastomosis (Figure 1).

To improve the analysis of preoperative and postoperative outcomes, the cerebral hemisphere was segmented into four regions: the anterior cerebral artery (ACA), the anterior part of the middle cerebral artery (ant-MCA), the posterior part of the middle cerebral artery (post-MCA), and the posterior cerebral artery (PCA). Changes in the sum of the cerebrovascular reserve (CVR) were compared between preoperative and postoperative conditions for each of these four regions. The main objective of this study was to demonstrate whether the ivy sign could serve as another indicator of hemodynamic changes, making the analysis of preoperative and postoperative HV (hyperintense vessels) the most important focus of this report. However, HV was not uniformly present across all vascular territories of the hemisphere. Therefore, we selected MR FLAIR image slices that showed typical HV and compared these slices with the corresponding SPECT images to assess CVR. To evaluate the usefulness of the ivy sign for follow-up, changes in the ivy sign on MR FLAIR images were compared with changes in CVR on SPECT images before and after STA-MCA anastomosis. We additionally annotated and compared anatomically matched slices in FLAIR and SPECT, highlighting the regions where hyperintense vessels were most prominent. The cortical regions with visible HV consistently overlapped with zones of reduced CVR on SPECT, indicating spatial concordance between structural and functional imaging. Clinically, patients presented with recurrent TIAs, cognitive slowing, or focal deficits refractory to medical therapy, prompting surgical revascularization. The improvement in the ivy sign was correlated with both imaging-based hemodynamic recovery and symptomatic relief.

Minimal and positive HVs were observed in 36 out of 49 patients (74%). Preoperative and postoperative MR FLAIR images were obtained at an average of 46.7 days (range: 8–99 days) and 196.6 days (range: 33–368 days) after surgery, respectively. A decrease in baseline cerebral blood flow (CBF) was noted in 92% of lesions, and 83% of areas with positive or minimal HV showed a decrease in CVR. In Moyamoya disease, positive HV and ivy scores were highest in the post-MCA region, while in steno-occlusive disease, the Spaghetti score was highest in the ant-MCA region.

After STA-MCA bypass surgery, 21 out of the 49 patients showed a disappearance of positive or minimal HV, and 11 patients showed a reduction in HV. In the Moyamoya disease group, HV improved as follows: from 10 to 5 in the ACA region, from 17 to 5 in the ant-MCA region, and from 24 to 11 in the post-MCA region. In the symptomatic steno-occlusive disease group, HV improved as follows: from 12 to 7 in the ACA region, from 23 to 10 in the ant-MCA region, from 7 to 4 in the post-MCA region, and from 3 to 2 in the PCA region. These results showed a statistically significant correlation (*p* < 0.05) when compared with CVR scores on SPECT images taken before and after surgery.

**Figure 2:**
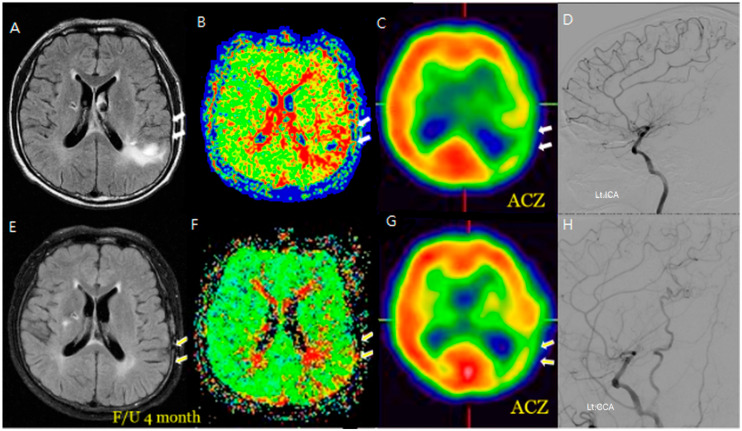
An M/58 symptomatic steno-occlusive patient (Rt. hemiparesis with Lt. M1 occlusion). Preoperative MR FLAIR, perfusion MR, and SPECT (**A**–**D**) show hyperintense vessels, indicating a decreased cerebral vascular reserve; a postoperative decrease or absence of hyperintense vessels reveals successful revascularization for decreasing HV after F/U; and a decrease in HV on the bypass-established hemisphere is associated with an improved hemodynamic status (**E**–**H**). Hyperintense vessels in preoperative FLAIR (**A**) coincide with a perfusion delay on SPECT (**C**). Postoperatively (**E**–**H**), the HV resolution matches with improved perfusion, demonstrating spatial and functional improvement.

**Figure 3:**
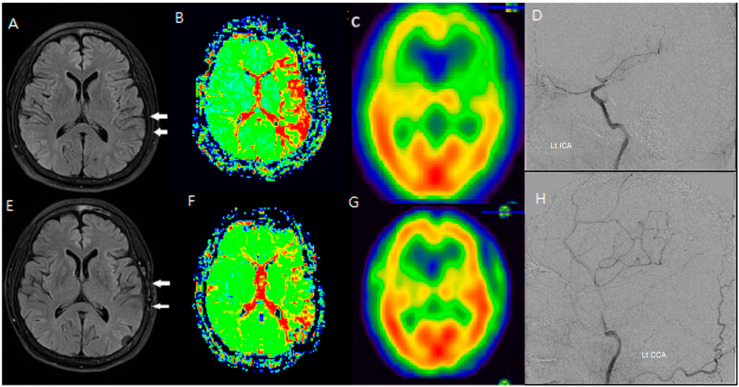
An F/66 patient with left distal ICA severe stenosis. (**A**–**D**) Preoperative MR FLAIR, perfusion MR, and SPECT images show prominent hyperintense vessels (white arrows) in cortical regions with a reduced CVR. (**E**–**H**) Postoperative imaging reveals the disappearance of HV and improvement in cerebral perfusion, indicating successful revascularization via STA-MCA bypass. HV regression corresponded to an increased CVR and reduced perfusion delay, reinforcing the functional significance of FLAIR findings.

Additionally, changes in the ivy sign on MR FLAIR images were compared with MR perfusion images. The degree of change in mean transit time (MTT) was greater in areas with positive or minimal HV compared to areas with negative HV. After STA-MCA bypass surgery, regions where HV decreased or disappeared showed improved hemodynamics, as reflected in both SPECT and MR perfusion imaging (Figure 2), and this correlation between HV regression and improved cerebral perfusion was similarly observed in a patient with distal ICA stenosis (Figure 3).

## 4. Discussion

Numerous studies have examined brain hemodynamic changes to date. Initially, these changes were measured based on CO_2_ reactivity, and SPECT became the standard method for assessing cerebral vascular reserve (CVR) by utilizing the metabolic effects of intravenous acetazolamide (ACZ) administration [5,6,7]. Additionally, the mean transit time (MTT) from MR perfusion has been valuable in evaluating hemodynamic changes in patients with Moyamoya disease and steno-occlusive disorders [2,8]. However, side-effects have been reported with SPECT and MR perfusion, including discomfort for patients, especially with T1-weighted MRI. As a result, extra caution is needed when administering ACZ to patients with severe ischemic symptoms. Furthermore, both MR perfusion and T1-weighted MRI require contrast agents, which means patients must undergo intravenous administration while fasting, requiring several hours of fasting prior to the procedure.

Recent studies suggest that the hypervascularity (HV) observed in MR FLAIR images offers new diagnostic value in detecting hemodynamic changes [2,9]. Several studies have indicated that HV on MR FLAIR images reflects the brain’s hemodynamic status in patients with Moyamoya disease and steno-occlusive disorders. We believe MR FLAIR may be more beneficial for follow-up evaluations compared to SPECT, perfusion MRI, or T1-weighted MRI, as it is less discomforting for patients with these conditions [5,10]. The MR FLAIR study has the advantage of being easily conducted in clinical settings. Compared to SPECT, MR perfusion, and T1-weighted MRI, MR FLAIR imaging can be completed in under ten minutes, has fewer side-effects, does not require fasting, and is a non-invasive method.

Postoperative FLAIR imaging frequently demonstrated a resolution or attenuation of the ivy sign. These changes are believed to reflect the normalization of leptomeningeal flow dynamics and the reduction in collateral recruitment following successful revascularization. In particular, the disappearance of hyperintense vessels in the post-MCA and ant-MCA regions aligns with improved CVR values in SPECT imaging. This correlation reinforces the utility of the FLAIR-based ivy sign as a practical marker of hemodynamic recovery after STA-MCA bypass in Moyamoya disease.

The ivy sign visualized on FLAIR imaging indirectly reflects impaired cerebral perfusion and collateralization efforts in response to arterial steno-occlusion. Although it does not directly quantify dynamic blood flow, its reduction postoperatively corresponds closely with improved CVR, supporting its utility as a surrogate marker for hemodynamic recovery.

The correlation between the distribution of the ivy sign and CVR reduction was consistent with the results of the SPECT study and FLAIR follow-up imaging.

The current sample has shown statistically significant results. By increasing the sample size, we can improve the reliability of the findings. We are currently in the process of collecting additional samples and plan to provide further updates in the future. Differences in surgeons can introduce various variables, including surgical outcomes. However, this study was conducted by a single experienced neurosurgeon and a single neurologist with expertise in neuroimaging, both from one center.

The ivy sign is the visualization of leptomeningeal collateral vessels, which form in response to cerebral hypoperfusion (insufficient blood flow). These vessels develop as an attempt to compensate for the stenosis or occlusion of major arteries, such as the internal carotid artery and middle cerebral artery. The presence of the ivy sign suggests that collateral circulation has developed in response to ischemia, but if CVR is reduced, this means the brain’s ability to increase blood flow and compensate for ischemia is failing, which can lead to more significant ischemic events like stroke or TIA (transient ischemic attacks). The ivy sign in Moyamoya disease is closely associated with CVR reduction and ischemic symptoms. It reflects the formation of collateral vessels as a compensatory mechanism for insufficient blood flow. While the ivy sign itself does not directly assess CVR, it can serve as a non-invasive indicator of reduced CVR and the ischemic burden of the disease. However, for a more accurate assessment of CVR, additional dynamic blood flow measurements and functional imaging techniques are still needed to complement the findings from ivy sign imaging. The ivy sign is a useful non-invasive marker to assess collateral circulation and can indirectly reflect the degree of CVR reduction in MMD. By using MRI or CT angiography, the ivy sign can be identified, and it can provide insights into the compensatory blood vessels’ development in response to ischemia. While the ivy sign itself does not directly measure blood flow, it indicates the brain’s attempt to develop collateral circulation in response to reduced flow. This can serve as an indirect measure of how CVR is functioning—if there is significant collateral formation, it suggests that CVR is impaired, as the brain is relying on these vessels to compensate for the lack of normal blood flow. Although the ivy sign cannot directly replace dynamic blood flow measurements or CVR testing (such as using functional MRI or PET to directly assess perfusion), it can still provide valuable information about the state of cerebral perfusion and the compensatory mechanisms in place, especially in situations where more invasive methods are not feasible. The correlation between the distribution of the ivy sign and CVR reduction was consistent with the results of the SPECT study and FLAIR follow-up imaging.

FLAIR imaging can provide reliable longitudinal follow-up for patients with Moyamoya disease or other cerebrovascular conditions without requiring invasive procedures, allowing for the safer and more frequent monitoring of disease progression. Although the average postoperative imaging interval was approximately 196 days, the wide range of follow-up durations (33–368 days) may affect the interpretation of HV resolution. A subgroup analysis or time-stratified evaluation would be beneficial in future research to address potential time-dependent effects on imaging findings. Since FLAIR imaging is non-invasive, it is easy to perform during long-term follow-up, and it is being used for evaluation alongside clinical symptom tracking in outpatient visits.

## 5. Limitations

Although our sample size was limited (n = 49), significant correlations between HV changes and hemodynamic improvements were clearly demonstrated, suggesting clinical relevance despite this limitation. Given the retrospective nature of our study, selection bias and uncontrolled confounding factors cannot be excluded, highlighting the need for prospective validation. In addition, potential confounding factors such as variations in the timing of follow-up imaging (33–368 days), antiplatelet medication use, and regional differences in vascular anatomy were not controlled in this study. These limitations may have influenced HV expression and perfusion measurements. Further large-scale, prospective studies are needed to validate the clinical utility of FLAIR imaging in the longitudinal monitoring of Moyamoya disease and symptomatic steno-occlusive cerebrovascular disorders.

## 6. Conclusions

The presence of HV on FLAIR images was observed in areas where both cerebral perfusion and CVR were reduced. Following STA-MCA anastomosis, the HV either diminished or disappeared in the affected hemisphere. Compared to traditional diagnostic tools like SPECT or MR perfusion, HV proves to be a valuable marker for detecting hemodynamic changes in the brain before and after surgery. Compared to traditional diagnostic tools like SPECT or MR perfusion, HV proves to be a valuable marker for detecting hemodynamic changes in the brain before and after surgery. Our findings indicate that HV is also an effective indicator for monitoring hemodynamic changes in adult patients. Additionally, the severity of HV was more pronounced in patients with greater symptom severity. Further research is needed to determine whether the ivy sign can also help assess improvements in hemodynamic status and evaluate the success of STA-MCA anastomosis in adult patients with Moyamoya disease and steno-occlusive disorders.

In conclusion, HV observed on FLAIR imaging correlates significantly with cerebral hemodynamics. Our findings indicate that changes in HV after STA-MCA bypass surgery reliably reflect hemodynamic improvements, suggesting its potential utility as a convenient, non-invasive imaging biomarker. Given that HV scoring on FLAIR imaging is qualitative and observer-dependent, future studies should pursue standardized, automated quantification techniques and multi-center prospective validation. These efforts will be crucial to improve reproducibility and enhance the translational applicability of HV as a non-invasive imaging biomarker in cerebrovascular revascularization.

## Figures and Tables

**Table 1:** Estimated regional HV scores and cerebrovascular reserve (CVR) before and after STA-MCA bypass.

Region	HV Score (Pre-Op)	HV Score (Post-Op)	CVR (Pre-Op) (%)	CVR (Post-Op) (%)
ACA	10	5	28.4	45.1
anterior MCA	17	5	25.6	43.2
posterior MCA	24	11	24.8	42.5
PCA	3	2	38.7	40.3

Note: HV scores represent the semi-quantitative grading of hyperintense vessels observed on FLAIR imaging. CVR values are estimated from cohort trends and are provided to illustrate regional hemodynamic changes.

## Data Availability

The data are not publicly available due to privacy or ethical restrictions.
